# Comparative Investigation of Untargeted and Targeted Metabolomics in Turmeric Dietary Supplements and Rhizomes

**DOI:** 10.3390/foods14010007

**Published:** 2024-12-24

**Authors:** Jashbir Singh, Fakir Shahidullah Tareq, Devanand L. Luthria

**Affiliations:** 1 Methods and Application of Food Composition Laboratory, Beltsville Human Nutrition Research Center, Agricultural Research Service, U.S. Department of Agriculture, Beltsville, MD 20705, USA; jashbir.singh@usda.gov (J.S.); fakir.tareq@usda.gov (F.S.T.); 2 Oak Ridge Institute for Science and Education (ORISE) Fellow, Oak Ridge, TN 37831, USA; 3 Department of Nutrition and Food Science, College of Agriculture & Natural Resources, University of Maryland, College Park, MD 20742, USA

**Keywords:** dietary supplements, curcuminoids (keto and enol forms), infrared and ultraviolet–visible spectroscopy, ultra-high performance liquid chromatography and gas chromatography coupled with mass spectrometry, multivariate analysis, disintegration study

## Abstract

In the present study, we analyzed the bioactive curcuminoids content in eight capsules (DS-1-DS-7 and DS-9), one tablet (DS-8), three ground turmeric samples (DS-10-DS-12), and three ground turmeric rhizomes (TR-1, TR-2, and TR-3). Initial screening with infrared and ultraviolet–visible spectroscopy coupled with a principal component analysis (PCA) revealed distinct differences between the samples analyzed. Hence, targeted and untargeted analyses were performed using ultra-high-performance liquid chromatography and gas chromatography coupled with mass spectrometry detections. The results show that the total curcuminoids content ranged from 1.3 to 69.8 mg/100 mg and the volatile component ranged from 0.7 to 9.1 mg/100 mg. The percentage ratio of the three prominent curcuminoids, bisdesmethoxycurcumin (BMC), desmethoxycurcumin (DMC), and curcumin (CUR), also varied remarkably compared to the expected ratio (BMC:DMC:CUR ratio of 1:2:6) described in the literature. The three prominent volatile compounds identified in most samples were ar-turmerone, turmerone, and curlone. The results demonstrated significant differences in the volatile compound levels among the DS and dried rhizome samples. The non-targeted analysis resulted in the identification of over 40 compounds, including bioactives such as piperine, phenolic acids, and amino acids. A disintegration study was performed on limited DS according to the United States Pharmacopeia protocol. The results reveal that all the selected DS samples passed the disintegration test. An analysis of curcuminoids from DS samples in neutral and acidic solutions demonstrated that all curcuminoids (BMC, DMC, and CUR) existed in the keto and enol forms and their concentrations changed with pH. This study will be of significant interest to manufacturers, consumers, and pharmacologists to accurately understand the bioactivities of three curcuminoids in different isomeric forms.

## 1. Introduction

*Curcuma longa* L., or turmeric, is a perennial herb belonging to the family Zingiberaceae, commonly used in Asian food curries and dietary supplements [1]. The United States Pharmacopeia (USP) defines “curcuminoids” as the sum of curcumin (CUR), desmethoxycurcumin (DMC), and bisdesmethoxycurcumin (BMC) as a partially purified natural complex of diarylheptanoid derivatives isolated from turmeric [1,2,3]. The global curcumin market for turmeric and dietary supplements is estimated at USD 155 million in 2024 and is expected to reach USD 240.68 million by 2029 [4]. Several turmeric-based products (capsules, tablets, dairy products, functional foods, and raw rhizome powder) are available in the market. They are susceptible to occasional contamination and adulteration with different substances [1,5,6]. Turmeric-based products either mixed with or substituted by other Curcuma species, starches, synthetic curcuminoids, or dyes can potentially cause issues related to human health [7,8]. For instance, the consumption of turmeric products contaminated with a yellow azo dye, metanil yellow, has been linked to neurotoxicity, while turmeric contaminated with lead chromate is associated with lead poisoning [9,10].

The rhizome of *C. longa* is commonly used for the preparation of dietary supplements (DS), which comprises mainly 3–5% diarylheptanoids (curcuminoids), and ~6% turmeric oil [11,12]. Curcuminoids (orange-yellow color) are key active non-volatile components of rhizome, while CUR, DMC, and BMC are major curcuminoids. Volatile turmeric oil is responsible for the aromatic rhizome flavor that is rich in sesquiterpenes. The major and most representative components of sesquiterpenes are aryl-, α-, and β-turmerones [13,14,15,16]. Both volatile and non-volatile components of turmeric rhizome are known for their various pharmacological properties. It is thus essential to evaluate the phytochemical composition of turmeric rhizomes for the quality control of DS on the market. Although numerous studies have been published on determining the curcuminoid ratio, limited studies are available on volatile analysis, non-targeted analysis, keto-enol forms of bioactive curcuminoids under different pH conditions, and disintegration studies.

Fourier transform infrared (FTIR) and near-infrared (NIR) spectroscopy techniques are valuable tools in identifying adulterant substances in nutraceuticals, functional foods, and spices [17,18]. Spectroscopic data coupled with chemometric modeling are powerful tools for determining the authenticity of samples. Several studies have been published on analyzing turmeric dietary supplements using spectroscopy techniques. For example, Shannon et al. reported the correct identification of typical turmeric samples (100%) and spiked samples (98.8%) using FTIR coupled with chemometric analysis [17]. Similarly, Thangavel et al. used FT-NIR along with a multivariate analysis to determine the curcumin, starch, and moisture contents in turmeric samples [18].

Previously, our lab developed a rapidly targeted UHPLC-MS method for separating curcuminoids and three adulterants, metanil yellow, Sudan I, and Sudan red G [5]. We also reported the roles of different solvents such as water, milk (homogenized, 2% reduced fat, low fat, fat free, soy, almond, coconut, and milkadamia), and different proportions of aqueous ethanol mixtures on the extractability of curcuminoids from turmeric powder [19]. In this study, we initially investigated the application of spectral fingerprinting methods to differentiate between commercial turmeric DS samples, including eight capsules (DS-1-DS-7 and DS-9), one tablet (DS-8), three ground turmeric samples (DS-10-DS-12), and three ground turmeric rhizomes (TR-1, TR-2, and TR-3) available from commercial sources. Furthermore, we performed targeted and non-targeted analyses to identify the bioactive curcuminoid concentration, ratios, and volatile components. In addition, we also performed disintegration studies on limited samples and investigated the influence of acidic pH on curcuminoids. This information will be significant to regulatory agencies, pharmacologists, manufacturers, and consumers.

## 2. Materials and Methods

### 2.1. Solvents and Materials

LC-MS-grade reagents (methanol, acetonitrile, formic acid, and water) were purchased from Fisher Scientific (Pittsburgh, PA, USA). GC-grade hexane was purchased from Fisher Scientific (Pittsburgh, PA, USA). Disposable 15 mL polypropylene centrifuge tubes used to extract metabolites from turmeric were purchased from Thermo-Scientific (Waltham, MA, USA). LC and GC vials were purchased from Fisher Scientific (Pittsburgh, PA, USA). Disposable syringe and polyvinylidene difluoride (PVDF) syringe filters with a pore size of 0.45 µm were purchased from National Scientific Company (Duluth, GA, USA). The standard curcuminoid samples were purchased from Toronto Research Chemicals Inc., Toronto, Ontario, Canada. The ar-tumarone standard was purchased from PhytoLab GmbH & Co. KG, Vestenbergsgreuth, Germany.

### 2.2. Experimental Samples

Commercial turmeric DS samples, including eight capsules (DS-1-DS-7 and DS-9), one tablet (DS-8), three ground turmeric samples (DS-10-DS-12), and three ground turmeric rhizomes (TR-1, TR-2, and TR-3), were purchased from commercial online and local grocery stores. Dried rhizome samples and tablets were ground using a typical coffee Mill grinder (Model 550K-Black, Muller, Austria) purchased from Amazom.com (Seattle, Washington, USA). The capsules were opened, and the ground powder was used for analysis. The polymeric capsule coverings were discarded.

### 2.3. FT-IR and NIR Data Analysis of DS and Rhizome Samples

Fourier transform infrared (FT-IR) and near-infrared (NIR) spectral fingerprints of turmeric DS and rhizome samples were analyzed using a Nicolet iS50 FT-IR with OMNIC-32 software (Thermo-Scientific, Waltham, MA, USA). Fine ground powder samples were separately placed in 4 mL glass vials and mixed thoroughly, and individual vials were analyzed in triplicate. Diffuse reflectance NIR and FT-IR spectra were collected from the ranges of 10,000–4000 cm^−1^ and 4000–400 cm^−1^, respectively, at a resolution of 4 cm^−1^ using an integrating sphere and ATR crystal, respectively. The spectral data were converted to Excel format. InGaAs 2.6 µm and DTGS ATR detectors were used for NIR and IR analyses, respectively. The spectral data were converted to Excel format, and multivariate analysis was performed using Solo software (release 7.8.2, Metlab version 7.9.0.529, Eigenvector Research, Inc., Manson, WA, USA).

### 2.4. Extraction and Analysis of Curcuminoids

Dietary supplements and turmeric rhizome powder (100 ± 0.2 mg) were extracted with 5 mL of 80% aqueous methanol under sonication (Advanced Sonic Processing Systems, Oxford, CT, USA) into 15 mL polypropylene centrifuge tubes for 30 min. Extracts were centrifuged at 1792× *g* for 15 min and filtered using 0.45 µm PVDF filter. The filtrate was transferred to 2 mL HPLC amber glass vials and analyzed by UHPLC-MS/MS analyses.

Ultraviolet–visible spectral analysis: Aliquots of methanolic extracts (100 µL) of DS and rhizomes were pipetted into a 96-well microplate reader (Molecular Devices, SPECTRAmax Plus 384, San Jose, CA, USA) equipped with a SoftMax Pro software version 5.2.0. The absorption intensity of the extracts and different concentrations of reference standards were collected at 420 nm.

UHPLC-DAD analysis of curcuminoids: UHPLC system (series 1290, Agilent Technologies, Santa Clara, CA, USA) consisting of a quaternary pump, autosampler, and diode array detector was used for curcuminoids analysis. Chromatographic separations were performed on C18 Agilent column (ZORBAX, Eclipse Plus, 2.1 × 50 mm, 1.8 µm, 1200 bar pressure limit, Agilent Technologies) using water (mobile phase A) and acetonitrile (mobile phase B) both acidified with 0.1% formic acid. The gradient program was set as follows: 35–50% phase B (0–11 min), and then 50–95% (11–12 min) and kept isocratic for 1 min, followed by 95–65% B (1 min) and kept isocratic for 3 min. The total run time was 16 min. The flow rate and the injection volume were maintained at 0.6 mL/min and 10 µL, respectively. Data were collected at 420 nm, and Open Lab Agilent software (M8301-61033, Agilent Technologies, Santa Clara, CA, USA) was used for data processing. Three standard curcuminoids (BMC, DMC, and CUR) were accurately weighed with an analytical balance and dissolved in methanol. The stock solution concentration was 100 μg/mL. Different concentrations (range of 0.8–50.0 µg/mL) were injected into the UHPLC-DAD, and calibration curves were prepared by plotting the peak area against the different concentrations.

### 2.5. GCMS Analysis of Hexane Extract

Extraction of non-polar compounds from dietary supplement and turmeric rhizome powder (100 ± 0.2 mg) was performed with 5 mL hexane. The solution mixture was sonicated for 15 min in a sonicator bath (Advanced Sonic Processing Systems, Oxford, CT, USA). Extracts were centrifuged at 1792× *g* for 15 min and filtered using a 0.45 µm PVDF filter. The filtrate was transferred to 2 mL amber color HPLC vials and analyzed by GC-MS.

GC-MS system performed the chromatographic separation of volatile compounds from hexane extracts from commercial dietary supplements and turmeric rhizomes (Agilent GC 6890, Santa Clara, CA, USA). An HP-5 MS capillary column 30 m in length with an internal diameter of 0.25 mm and a film thickness of 0.25 µm was used (Agilent, Santa Clara, CA, USA). The injection and mass transfer line temperatures were set at 260 °C and 250 °C, respectively. The amount injected into the GCMS system in spitless mode was 1 µL. Helium was used as a carrier gas at a flow rate of 1.0 mL/min. The temperature program was set as follows: the initial oven temperature was kept at 50 °C for 2 min and then gradually increased to 100 °C at a rate of 5 °C/min. The temperature was maintained at 100 °C for two additional minutes. The temperature again increased from 100 °C to 250 °C at the rate of 10 °C/min and held at 250 °C for two additional minutes. Total run time was 31 min. A single quadrupole mass spectrometer was operated at 70 eV with the electron ionization (EI) source kept at 260 °C. A total of 781 scans per second were recorded over the mass range of *m*/*z* 50–1000. The identification of volatile compounds was achieved using the mass spectra database of the National Institute of Standards and Technology (Ver. NIST MS search 2.0) and with data in the literature.

### 2.6. Untargeted Metabolomic Analysis

Extracts obtained from turmeric DS and dried rhizomes were analyzed with the Vanquish UHPLC system coupled with a diode array detector (DAD), charged aerosol detector (CAD), and an Exploris 240 mass high-resolution mass spectrometer (Thermo Scientific, Waltham, MA, USA). The UHPLC conditions for chromatographic separation are described in Section 2.7, except the injection volume was 5 µL. The ESI-MS conditions were as follows: the mass range was set in a range of 100–1000 *m*/*z*, and sheath gas, auxiliary, and sweep gas were set at 50, 10, and 1 (arbitrary units), respectively. Capillary and vaporizer temperatures were set at 320 °C and 350 °C, respectively, while spray voltage was set at 3.5 kV. The full scan mass spectra and data-dependent acquisition (DDA) events were acquired at a resolving power of 60,000. For MSn activation, an isolation width of 1 amu; a maximum ion injection time of 100 ms; stepped collision energy values of 30, 50, and 150 eV; and an activation time of 10 ms were used. High-resolution mass data were acquired in both positive and negative ionization modes. The software package Xcalibur 4.4 (Thermo Scientific, Waltham, MA, USA) was used to analyze the mass spectral data.

### 2.7. Disintegration Study of Selected Dietary Supplements

Dietary supplement disintegration was performed according to the USP 39 general chapters 2040 and 701 for immediate release DS forms [20,21,22]. Briefly, six capsules (DS-3 and DS-9) were individually immersed in the 0.05 M acetate buffer media, and six tablets (DS-8) were in the water and agitated media for 30 min. Tablets were immersed in water, and capsules with hard shells (gelatin or non-gelatin) were immersed in acetate buffer (pH 4.5 at 37 °C). Apparatus A was used for units <18 mm in length, and Apparatus B was used for units >18 mm in length (Vankel VK 100, Varian Inc., Cary, NC, USA). No disks were added to the disintegration tubes.

### 2.8. Impact of Acidic pH on Turmeric Dietary Supplement

Analysis of the curcuminoid profile from a single dietary supplement was performed to investigate the changes under acidic pH. This was achieved via a release study performed using 0.1 N HCl solution. Approximately, 250 ± 1 mL of 0.1 N HCl solution was taken from a 500 mL beaker placed on a Corning hot plate magnetic stirrer (PC-351, Corning, New York, NY, USA). A dietary supplement capsule was added, the stirring speed was set at ~60 rpm (dial setting between 1 and 2), and the temperature was maintained at 37 °C (dial setting just over 2) according to the manufacturer manual. The temperature of the bath was checked manually with a thermometer. Sample (1 mL) was collected at 0 min, 15 min, 30 min, 45 min, 60 min, 90 min, and 120 min. One milliliter of methanol (100%) was added to the collected sample, and the mixture was vortexed for 1 min and centrifuged. Samples were filtered through a 0.45 µm PVDF syringe filter before UHPLC analysis.

### 2.9. Data Processing

For quantification, the area of each curcuminoid was collected using the LC OpenLab program from Agilent Technologies (M8301-61033, Agilent Technologies, Santa Clara, CA, USA) was used to analyze and identify peaks in the chromatogram. Compound Discoverer (Version 3.3, Thermo Scientific, Waltham, MA, USA) program was used to collect the maximum features from the raw HRMS data. The workflow of the program to analyze the raw data includes the extraction of the MS spectrum, align retention time, the detection of compounds, group compounds, the deconvolution of the overlapping ions based on their isotope patterns (fill gaps), and the prediction of the composition using existing database search (mzCloud, Chemspider, and mzLogic) and in-house library search. The resulting areas of each sample were exported to Microsoft Excel 365 (Microsoft, Redmond, WA, USA) in triplicate for analysis.

### 2.10. Statistical Analysis

Principal component analysis (PCA) of curcuminoids and volatile compounds was performed using Pearson’s correlation method and one-way analysis of variance (ANOVA) with XLSTAT software (version 2023.2.0; Addinsoft, Paris, France). Tukey’s HSD test observed significant differences between means at a 5% probability level (*p* ≤ 0.05).

## 3. Results and Discussion

### 3.1. Principal Component Analysis of IR and NIR Spectral Fingerprints

**Figure 1:**
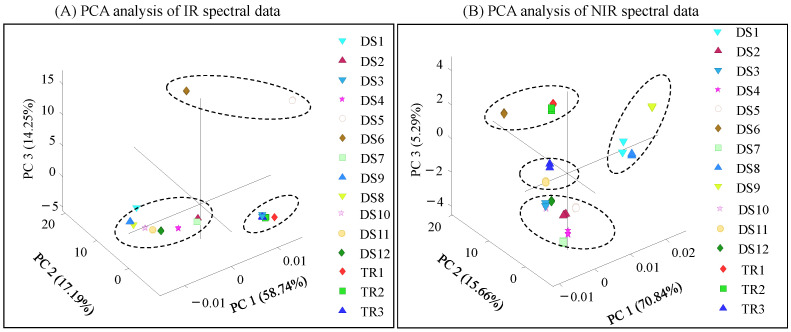
Principal component analysis of infrared (**A**) and near-infrared (**B**) spectral data obtained from different dietary supplements, powdered turmeric, and ground turmeric rhizome. Commercial turmeric DS samples included eight capsules (DS-1-DS-7 and DS-9), one tablet (DS-8), three ground turmeric samples (DS-10-DS-12), and three ground turmeric rhizomes (TR-1, TR-2, and TR-3).

FT-IR and NIR spectroscopy techniques are widely used in different research areas and industries due to their speed, high precision, and potential to perform a non-destructive analysis in a cost-effective manner. In the present study, we used FT-IR and NIR spectral data combined with PCA to understand the compositional differences between eight capsules (DS-1-DS-7 and DS-9), one tablet (DS-8), three ground turmeric samples (DS-10-DS-12), and three ground turmeric rhizomes (TR-1, TR-2, and TR-3) (Figure 1). A PCA of the FT-IR spectral data showed three distinct clusters. Cluster 1, which includes turmeric rhizomes (TR1-TR3) and DS-3; cluster 2, which includes DS-5 and DS-6; and cluster 3, which includes DS-1, DS-2, DS-4, DS-7, DS-8, DS-9, DS-10, DS-11, and DS-12. The first, second, and third principal components showed 90.2% of the total variation (Figure 1A). The PCA of the NIR spectral data also showed four clusters. TR1 and TR2 were clustered together with DS-6, while TR3 was clustered with DS-11. However, DS-1, DS-8, and DS-9 were clustered together and showed distinct separation from the other analyzed DS samples (2, 3, 4, 5, 6, 7, 10, 11, and 12). The first, second, and third principal components explained 91.8% of the total variation (Figure 1B).

These results demonstrate that DS and raw turmeric rhizomes were significantly influenced by the chemical composition and the capsule matrix used for dietary supplement formulation. In a published study, the authors used FT-IR spectral methods to identify turmeric samples adulterated with starch in commercially available turmeric powder [17]. Similarly, Kar et al. used the FT-NIR-PLSR (Fourier transform near-infrared–partial least square regression) model to determine adulteration in turmeric powder. They found that starch adulteration in commercial samples was in the range of 1–30% (*w*/*w*) [23]. In our study, IR and NIR showed significant differences in the clustering patterns between dietary supplements and rhizome samples. Hence, we performed UV–visible screening of all DS samples and three rhizome samples.

### 3.2. Ultraviolet–Visible (UV–Vis) Absorption Spectroscopy Screening and Targeted UHPLC-DAD Analysis of Curcuminoids from Dietary Supplements and Turmeric Rhizomes

The UV spectrum of curcuminoids showed absorption maximum at 420 nm due to the presence of chromophore 1,7-bis(4-hydroxy-3-methoxyphenyl)-1,6-heptadiene-3,5-dione) in their chemical structure, which could be used as a screening tool to discriminate samples based on total curcuminoid contents. All DS samples were extracted uniformly with methanol and screened in triplicate at a fixed 420 nm wavelength in 96-well plates. The results show that the absorption maximum at 420 nm varied between 0.15 and 0.83 ODU. A calibration curve was constructed with the authentic curcuminoid standard. The concentrations were calculated based on the absorption maxima. The results were correlated with a detailed UHPLC-DAD analysis.

The UHPLC-DAD analysis showed three major peaks: bisdemethoxycurcumin (BMC), demethoxycurcumin (DMC), and curcumin (CUR). The identification and quantification of each compound were confirmed with a mass spectroscopy analysis. The mass spectra obtained in negative ionization mode of peak eluting at a retention time (t_R_) of 8.56 min showed a molecular ion at *m*/*z* 307.0978 [M-H]^−^, and its major product ions at *m*/*z* 187.0627 were identified as BMC. Similarly, a peak eluting at t_R_ 9.19 min displayed a molecular ion peak at *m*/*z* 337.1083 [M-H]^−^, and their product ions at *m*/*z* 217.0507, 173.0469, and 134.0360 were identified as DMC. The major peak (t_R_ 9.82 min) with molecular mass at *m*/*z* 367.1188 [M-H]^−^ and the product ions at *m*/*z* 217.0507 were identified as CUR. The presence of fragmentation ions at *m*/*z* 217.0507 in the mass spectra of DMC [M-H-120]^−^ and CUR [M-H-150]^−^ represents the presence of one or two methoxyl groups, respectively. Our findings are in good agreement with the previous literature [24,25].

A significant difference (*p* <  0.05) was observed in the levels of curcuminoids among the different dietary supplement and rhizome samples. The levels of individual and total curcuminoids in all samples are presented in Table 1. The concentration of total curcuminoids ranged between 1.4 ± 0.0 mg/100 mg (DS-3) and 69.8 ± 3.2 (DS-9) mg/100 mg on a dry weight basis. Curcumin was the most predominant curcuminoid in all samples. The levels of CUR were also significantly higher in DS-8 (31.0 ± 2.2 mg/100 mg DW) and DS-9 (51.2 ± 2.2 mg/100 g DW), while lower levels were observed in DS-3 (0.8 ± 0.0 mg/100 mg DW) and DS-10 (0.8 ± 0.1 mg/100 mg DW). The levels of DMC were higher than BMC in samples DS1-DS9 except for the two dietary supplement samples (DS10-DS11) and the three rhizomes (TR1-TR3). Interestingly, DS12 showed very similar amounts of BMC and DMC. The percentage ratio of the individual curcuminoids BMC, DMC, and CUR also varied remarkably, as shown in Figure S1, compared to the expected ratio (BMC:DMC:CUR ratio of 1:2:6) described in the literature [1,15,26]. In DS8, significantly higher levels of curcumin were present compared to DMC and BMC (31.0:5.6:1.3). However, in DS11, the ratio of CUR:DMC:BMC was 1.4:0.9:1.3. The variations observed in the total curcuminoid content and the ratios of the individual curcuminoids can be attributed to multiple factors, such as processing, variations in cultivars, growing locations, etc.

There was a high correlation (R2 = 0.99) between the UV–vis and UHPLC-DAD data analysis, suggesting that UV–vis spectroscopy can be used as a quick cost-effective screening tool for the determination of the curcuminoid content with a careful understanding of the other components present in the matrix. The limitation of the UV–vis spectroscopy screening method is that no ratio information on individual curcuminoids can be obtained. The presence of synthetic curcumin in the sample can be determined by comparing the curcumin-to-curcuminoid ratio and a threshold of 85.44% for the relative curcumin value calculated using USP and AOAC criteria [22]. In our study, no DS sample showed the presence of spiked curcumin (Table 1). In a recent study, You et al. showed that 5 supplements out of 14 samples showed the presence of synthetic curcumin [1].

**Figure 2:**
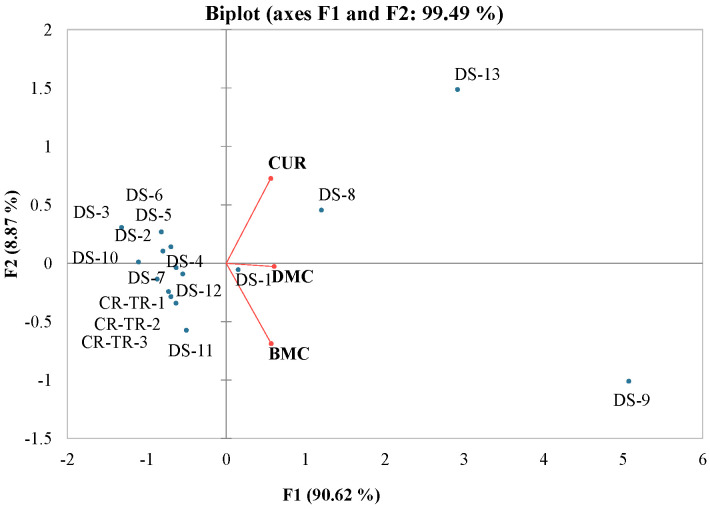
Principal component analysis of bisdemethoxycurcumin (BMC), demethoxycurcumin (DMC), and curcumin (CUR) obtained from different dietary supplements (eight capsules (DS-1-DS-7 and DS-9), one tablet (DS-8)), powdered turmeric (DS-10-DS-12), and ground turmeric rhizomes (TR-1, TR-2, and TR-3).

A principal component analysis (PCA) was conducted to visualize curcuminoid concentrations among the analyzed samples. The principal component F1 elucidated 90.6% of the data variation and only differentiated curcuminoids on the F1 component. All the analyzed DS samples and turmeric rhizomes were also differentiated on F1 components except DS-11, which was explained by the F2 component. Overall, the PCA demonstrated 99.5% of the total variation in the first two PC components (Figure 2).

### 3.3. GCMS Analysis

Three prominent volatile compounds, ar-turmerone, turmerone, and curlone, were identified in all turmeric dietary supplements (DS) except DS-9 in which turmerone was not detected. A typical GCMS chromatogram of the three volatile compounds identified from the dietary supplements, ground turmeric, and rhizome is presented in Figure S2. The results demonstrate significant differences in the volatile compound levels among the DS and dried rhizome samples. Significantly higher levels (three times higher) of the three prominent volatile compounds were found in DS-11, while the lowest levels were observed in DS-9. No significant difference was observed among the turmeric rhizomes (Table 2).

Moreover, in the rhizome samples, we also identified α-curcumene, zingiberene, β-bisabolene, and β-sesquiphellandrene, which were absent in the DS samples. This can be attributed to processing and storage conditions which might result in a significant loss of volatile compounds. These findings are in good agreement with previous reports in which *ar*-turmerone was identified as a major sesquiterpenoid in turmeric dietary supplements [1,15]. In another report, higher levels of *ar*-turmerone were observed in dried turmeric rhizomes, but their content was lower than that in fresh rhizomes [27].

**Figure 3:**
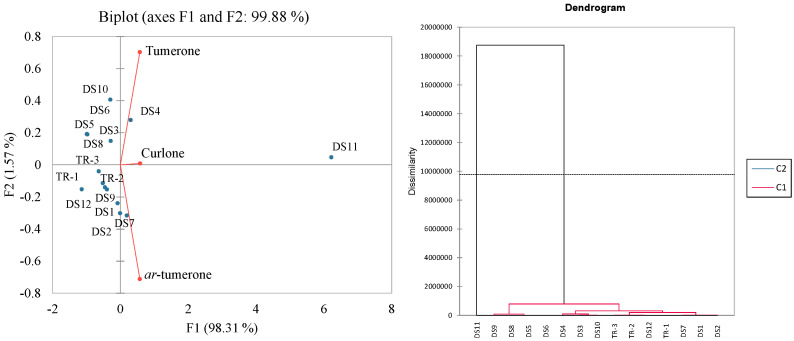
Principal component analysis and Hierarchical Cluster Analysis of volatile compounds (*ar*-tumerone, tumerone, and curlone) obtained from commercial turmeric DS samples: eight capsules (DS-1-DS-7 and DS-9), one tablet (DS-8), three ground turmeric samples (DS-10-DS-12), and three ground turmeric rhizomes (TR-1, TR-2, and TR-3).

PCA and HCA (Hierarchical Cluster Analysis) were conducted to visualize the variations in the volatile compounds among the DS and dried rhizome samples (Figure 3). The results show that the DS and dried rhizome samples were easily differentiated from each other. It was also observed that DS-11 had a high level of volatile compounds compared to all other samples. The volatile compounds *ar*-tumerone, tumerone, and curlone were differentiated based on component F1 only. All dried rhizome samples (TR1-TR3) and DS samples DS-3, DS-4, DS-5, DS-6, DS-8, DS-9, DS-11, and DS-12 were differentiated by principal component F1, while DS samples DS-1, DS-2, DS-7, and DS-10 were differentiated by F2. Overall, the first and second components explained 99.9% of the total variances.

### 3.4. Untargeted Metabolomics Analysis Using Compound Discoverer Software

An untargeted metabolism coupled with the Compound Discoverer software was used for the putative identification of untargeted metabolites. More than 400 metabolites were putatively identified using multiple databases such as mzCloud, ChemSpider, Metabolika, an in-house mass list, and other online available databases in positive and negative ionization modes. Among them, 32 metabolites were confirmed in positive ionization modes and 18 metabolites in negative ionization modes by MS/MS fragments and the available data in the literature (Table 3).

Keto-enol tautomerism is pH-dependent; the keto form is predominant in acidic or neutral conditions, while the enol form is dominant under basic conditions [28,29]. The published literature identified 89 curcuminoids, including keto-enol forms in turmeric, using a UHPLC-QTOF-MS/MS analysis [24]. The enol form of curcuminoids has a longer retention time than the keto form. Similar results were observed in our study; we identified three keto and three enol curcuminoid forms. We also identified piperine in DS-4, DS-7, and DS-9, while a prominent essential oil, ar-tumerone, was also found in all analyzed samples. The published literature revealed that piperine enhanced the bioavailability of curcumin by limiting the extent of curcumin glucuronidation by inhibiting the enzyme UDP-glucuronyltransferase in the liver [30,31]. They found a 97-fold enhancement in curcumin bioavailability compared to pure curcumin after 6 h of piperine administration [31]. Clinical studies also showed the effective role of the co-administration of curcumin and piperine in improving human health and protecting against non-fatty liver disease, type 2 diabetes mellitus, certain types of tumors, obesity, and other metabolic diseases [32,33,34,35].

**Figure 4:**
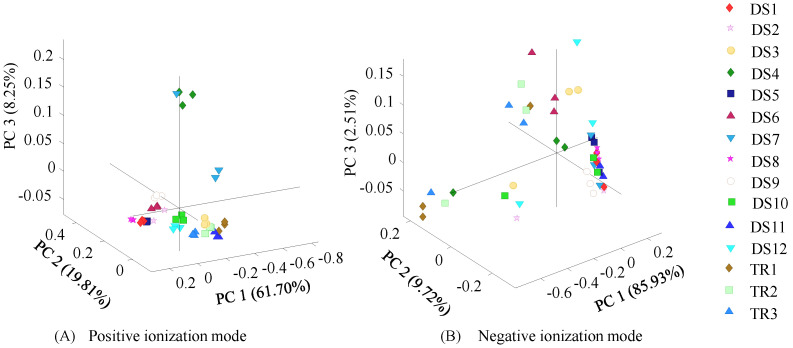
PCA of untargeted metabolite high-resolution mass data of different dietary supplements, powdered turmeric, and ground turmeric rhizomes. Commercial turmeric DS samples included eight capsules (DS-1-DS-7 and DS-9), one tablet (DS-8), three ground turmeric samples (DS-10-DS-12), and three ground turmeric rhizomes (TR-1, TR-2, and TR-3).

A PCA was conducted to amplify and detect the subtle differences between DS and rhizomes based on identified metabolites (Figure 4). A PCA of mass data obtained in positive ionization mode showed a distinct separation of DS-4 and DS-7 from the other analyzed samples. The first (PC1), second (PC2), and third (PC3) principal components explained 61.7%, 19.8%, and 8.3% of the variance in positive ionization mode, respectively. The loading plots show that PCA differentiates according to the curcuminoid keto and enol isomeric forms, alpha-curcumene, and caryophyllene oxide in both the first and second principal components, while piperine and curcuminoids isomers are differentiated in PC3. Similarly, the results obtained from the PCA of the mass data obtained in negative ionization mode display separation among the rhizome and DS samples. PC1, PC2, and PC3 explain 85.9%, 9.7%, and 2.5% of the total variance in negative ionization mode.

### 3.5. Quantification Variability and Labeling

Accurate labeling of dietary supplements is important for regulatory compliance and consumers’ health. Our results demonstrate significant variation in the labeling accuracy of commercial products. The quantity of curcuminoids in DS-2, DS-4, and DS-9 were within 10% of the label range, while the detected amounts in DS-1, DS-5, and DS-8 (approximately 24.7%, 42.0%, and 72.0%, respectively) were significantly below the label claims. Interestingly, no claims on the total curcuminoid content were depicted in the labels of DS-3, DS-6, and DS-7. The rhizome samples, TR-1, TR-2, and TR-3, had curcuminoid contents according to the published literature or within the range of accepted values. Recently, You et al. 2022, reported variation in the label accuracy, and only 4 out of the 14 samples analyzed supported authentic label claims [1].

### 3.6. Disintegration Analysis on Limited Dietary Supplements and Effect on Curcuminoids in Acidic pH Conditions

The disintegration of tablets and capsules is the essential process for the determination of the release of active ingredients. According to the United States Pharmacopeia (USP), complete disintegration occurs when a tablet or capsule has disintegrated into a soft mass with no firm core and no residue remains on the screen of the test apparatus (except fragments of the insoluble coating or capsule shell) after 30 min [20,21,22]. Our results show that all the selected DS samples passed according to USP standards (Table 4).

**Figure 5:**
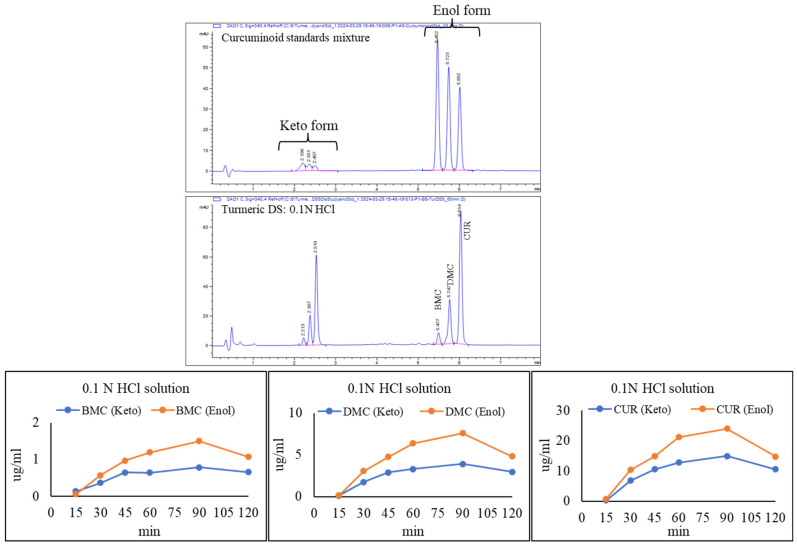
HPLC chromatograms (wavelength 340 nm) and release study of three curcuminoids (bisdesmethoxycurcumin (BMC), desmethoxycurcumin (DMC), and curcumin (CUR)) in turmeric dietary supplement (capsule) in 0.1 N HCl solution.

We also investigated the effect of acidic pH on curcuminoids from a single dietary supplement capsule in 0.1 N HCl solution. Chromatograms and data were collected at 340 nm to observe the changes in the keto-enol forms of curcuminoids (Figure 5). The results demonstrate that the release of curcuminoids (keto and enol forms of BMC, DMC, and CUR) from the capsule increased with time until 90 min. After that, both forms showed a decrease in their levels until 120 min.

The published literature reports that the decomposition of curcuminoids is pH-dependent [36,37,38]. In acidic conditions, the decomposition of curcumin is lower (<20% decomposition after 1 h) compared to the basic–neutral solution [37,39,40]. The higher stability of curcuminoids in acidic conditions may be due to a conjugated diene structure. In a higher pH, the chemical structure was destroyed due to the removal of the proton group from the phenolic group. Puglisi and co-authors reported that the keto-enol tautomers of curcuminoids depend on the pH, solvent, and temperature [36]. In acidic conditions, curcuminoids act as proton donors and are mainly present in keto form [37]. The amount of keto-enol moiety plays a significant role in curcuminoids’ physicochemical properties and antioxidant activities [41]. The keto form is mainly responsible for curcuminoids’ strong antioxidant activity [36].

## 4. Conclusions

Spectroscopy techniques, including FTIR, NIR, LC-MS, GCMS, MS-spectral database/literature search, and chemometric treatment, enabled us to establish the significant variability in turmeric dietary supplements and rhizomes. Quantitation and the proportions of three curcuminoids (BMC, DMC, and CUR) and three prominent volatile compounds (ar-turmerone, turmerone, and curlone) showed significant variations in their levels among the different DS samples and rhizomes. The dietary supplements DS-2, DS-4, and DS-9 were within the 10% range of the label claim. No adulteration of the dietary supplements with synthetic dyes was observed. All the selected DS samples passed the disintegration analysis test. A release study of DS samples in an acidic solution showed that curcuminoids were present in keto-enol forms, and their levels initially increased until 90 min and then decreased. Overall, to accurately evaluate the role of bioactive curcuminoids in turmeric, it is essential to analyze and understand the bioactivities of different isomeric forms. This will enable researchers to assess the role of curcuminoids as it relates to health and nutrition.

## Figures and Tables

**Table 1:** Levels of Bis-desmethoxycurcumin (BMC), desmethoxycurcumin (DMC), and curcumin (CUR) in different dietary supplements, powdered turmeric, and ground turmeric rhizome analyzed by GCMS. Commercial turmeric DS samples included eight capsules (DS-1-DS-7 and DS-9), one tablet (DS-8), three ground turmeric samples (DS-10-DS-12), and three ground turmeric rhizomes (TR-1, TR-2, and TR-3). Label claims regarding the amount of curcuminoids present in the dietary supplement. Data represent mean ± SE and mg/100 mg. Presence of synthetic curcumin was speculated according to percentage of curcumin to total curcuminoid was found to be more than 85.44%.

Group	BMC (mg/100 mg)	DMC (mg/100 mg)	CUR (mg/100 mg)	Total Curcuminoids (mg/100 mg)	Curcumin-Total Curcuminoid Ratio (%)	Amount per Serving	Label Claim/Serving (mg)	Calculated Total Curcuminoid Content (mg/Serving)	Sample Type/Description	Label Claim in % (mg/Serving)
DS-1	1.1 ± 0.1	3.2 ± 0.4	12.9 ± 2.4	17.4 ± 2.9	74.8	1 capsule	500	123.5	Capsule/turmeric extract rhizome	24.7
DS-2	0.6 ± 0.0	1.2 ± 0.0	4.7 ± 0.12	6.5 ± 0.2	72.3	1 capsule	47.5	44.6	Capsule/turmeric extract rhizome and powder	94
DS-3	0.2 ± 0.0	0.9 ± 0.0	0.8 ± 0.0	1.4 ± 0.0	61.4	2 capsules	n.a.	23.0	Capsule/turmeric powder	No label claim
DS-4	0.9 ± 0.0	1.4 ± 0.0	5.5 ± 0.1	7.8 ± 0.2	70.1	3 capsules	150	164.1	Capsule/turmeric extract rhizome and bioperine	109
DS-5	0.6 ± 0.0	1.6 ± 0.1	5.8 ± 0.5	8.0 ± 0.7	73.0	2 capsules	180 to 220	84.1	Capsule/turmeric extract rhizome	42
DS-6	0.4 ± 0.0	1.3 ± 0.0	6.2 ± 0.2	7.9 ± 0.2	77.5	2 capsules	n.a.	75.8	Capsule/turmeric rhizome extrcat (Phytosome)	No label claim
DS-7	0.8 ± 0.0	1.3 ± 0.1	5.13 ± 0.3	7.3 ± 0.4	70.5	2 capsules	n.a.	104.3	Capsule/turmeric extract rhizome and bioperine	No label claim
DS-8	1.3 ± 0.1	5.6 ± 0.3	31.0 ± 2.2	37.9 ± 2.5	81.8	1 tablet	475	345.9	Tablet/turmeric extract rhizome	72
DS-9	4.3 ± 0.4	14.3 ± 0.7	51.2 ± 2.2	69.8 ± 3.2	73.4	2 capsules	950	945.7	Capsule/turmeric extract rhizome and bioperine	99
DS-10	0.5 ± 0.0	0.4 ± 0.0	0.8 ± 0.1	1.7 ± 0.1	45.8	n.a.	n.a.	n.a.	Powder	n.a.
DS-11	1.3 ± 0.0	0.9 ± 0.0	1.4 ± 0.0	3.6 ± 0.1	37.8	n.a.	n.a.	n.a.	Powder	n.a.
DS-12	0.7 ± 0.0	0.7 ± 0.0	1.7 ± 0.0	3.2 ± 0.1	52.9	n.a.	n.a.	n.a.	Powder	n.a.
TR-1	1.1 ± 0.1	0.9 ± 0.0	1.8 ± 0.1	3.8 ± 0.2	47.6	n.a.	n.a.	n.a.	Rhizome	n.a.
TR-2	1.0 ± 0.1	0.8 ± 0.0	1.7 ± 0.1	3.6 ± 0.2	47.3	n.a.	n.a.	n.a.	Rhizome	n.a.
TR-3	1.0 ± 0.1	0.8 ± 0.0	1.8 ± 0.1	3.6 ± 0.2	49.8	n.a.	n.a.	n.a.	Rhizome	n.a.

**Table 2:** Levels of volatile compounds in hexane extracts of different dietary supplements, powdered turmeric, and ground turmeric rhizomes analyzed by GCMS. Commercial turmeric DS samples included eight capsules (DS-1-DS-7 and DS-9), one tablet (DS-8), three ground turmeric samples (DS-10-DS-12), and three ground turmeric rhizomes (TR-1, TR-2, and TR-3). Data represent mean ± SD (standard deviation) and mg/100 mg.

Group	*ar*-Tumerone	Tumerone	Curlone
DS-1	0.83 ± 0.24	0.45 ± 0.05	0.63 ± 0.09
DS-2	0.92 ± 0.19	0.49 ± 0.04	0.60 ± 0.02
DS-3	0.6 ± 0.01	0.62 ± 0.01	0.53 ± 0.00
DS-4	0.73 ± 0.10	0.94 ± 0.16	0.73 ± 0.10
DS-5	0.32 ± 0.00	0.32 ± 0.00	0.32 ± 0.01
DS-6	0.33 ± 0.00	0.32 ± 0.00	0.33 ± 0.00
DS-7	1.00 ± 0.01	0.58 ± 0.02	0.66 ± 0.03
DS-8	0.33 ± 0.00	0.32 ± 0.00	0.33 ± 0.01
DS-9	0.39 ± 0.00	nd ± nd	0.34 ± 0.00
DS-10	0.47 ± 0.01	0.76 ± 0.04	0.53 ± 0.02
DS-11	3.12 ± 0.82	3.70 ± 1.54	2.29 ± 0.39
DS-12	0.68 ± 0.08	0.38 ± 0.02	0.47 ± 0.03
TR-1	0.65 ± 0.08	0.38 ± 0.02	0.44 ± 0.01
TR-2	0.72 ± 0.10	0.41 ± 0.02	0.47 ± 0.12
TR-3	0.57 ± 0.09	0.36 ± 0.01	0.41 ± 0.03

**Table 3:** Putative identification of untargeted metabolites in turmeric supplements and roots using UHPLC-HRMS data analysis acquired in positive and negative ionization modes.

S.No.	Compound	Formula	t*_R_* (min)	*m*/*z* [M]	[M + H]^+^	[M + H]^−^
1	Choline	C5 H13 N O	0.858	103.09975	104.10657	-
2	Maltose	C12 H22 O11	0.861	342.11619	343.1234	-
3	L-Glutamic acid	C5 H9 N O4	0.862	147.05323	148.0605	-
4	Sucrose	C12 H22 O11	0.867	342.1177	-	341.11
5	Isocitric acid	C6 H8 O7	1.107	192.02784	-	191.021
6	Chlorogenic acid	C16 H18 O9	1.15	354.09705	-	353.09
7	Leucine	C6 H13 N O2	1.164	131.09468	132.10196	-
8	Ferulic acid	C10 H10 O4	1.395	194.05877	-	193.052
9	4-Hydroxybenzaldehyde	C7 H6 O2	1.489	122.03714	-	121.03
10	Nonanedioic acid	C9 H16 O4	1.523	188.10561	-	187.098
11	BMC (keto)	C19 H16 O4	3.043	308.10492	309.11218	307.099
12	DMC (keto)	C20 H18 O5	3.349	338.1155	339.1153	337.11
13	Curcumin (keto)	C21 H20 O6	3.643	368.12601	369.13325	367.12
14	6,8,10,12-pentadecatetraenal	C15 H22 O	7.65	218.16704	219.1743	-
15	L-(-)-Carvone	C10 H14 O	8.643	150.10453	151.1118	-
16	p-tert-Butylbenzoic acid	C11 H14 O2	8.652	178.09939	179.1066	-
17	Piperine	C17 H19 N O3	8.84	285.13644	286.1437	-
18	BMC (enol)	C19 H16 O4	8.688	308.10482	309.11209	307.1
19	4-Hydroxybenzaldehyde	C7 H6 O2	8.782	122.03685	-	121.03
20	DMC (enol)	C20 H18 O5	9.333	338.11535	339.1153	337.108
21	4-Hydroxybenzaldehyde	C7 H6 O2	9.397	122.03686	-	121.03
22	Hydrocinnamic acid	C9 H10 O2	9.95	150.06832	-	149.061
23	Curcumin (enol)	C21 H20 O6	9.965	368.12585	369.1258	367.119
24	2,4-Dimethylbenzaldehyde	C9 H10 O	11.803	134.07321	135.0804	-
25	(+)-ar-turmerone	C15 H20 O	12.059	216.15139	217.1586	-
26	Lysophosphatidyl ethanolamine LPE (18:2)	C24 H40 N5 O3 P	13.587	477.28561	478.2928	-
27	Lysophosphatidylcholine (LPC (18:2)	C27 H46 N5 O3 P	13.606	519.33253	520.3398	-
28	N-stearoyl glutamic acid	C23 H43 N O5	13.705	413.3142	-	412.307
29	Hexadecanamide	C16 H33 N O	13.725	255.25621	256.2634	-
30	Tetrahydroxycurcumin	C30 H40 O2	13.861	432.3029	433.3101	-
31	Poly-L-aspartic acid	C4 H7 N O4	13.87	133.03755	134.0448	-
32	6-Prenylchrysoeriol	C21 H20 O6	13.902	368.12608	-	367.119
33	Linoleyl hydroxamic acid	C18 H33 N O2	13.967	295.25111	296.2583	-
34	Octa-2,4,6-triynoic acid	C4 H11 N4 P	14.051	146.07184	147.1168	-
35	Alpha-curcumene	C15 H22	14.065	202.17208	203.1793	-
36	Didesmethyl tocotrienol	C25 H36 O2	14.263	368.27154	369.2788	-
37	LysophosphatidylinositolLPI (16:0)	C37 H40 N4 O2	14.382	572.31415	573.3214	-
38	(-)-Caryophyllene oxide	C15 H24 O	14.451	220.18267	221.1899	-
39	Alpha-Farnesene	C15 H24	14.513	204.18777	205.19504	-
40	Phenol, 4-cyclohexyl-	C12 H16 O	14.581	176.12014	177.1273	-
41	4-Phenyl Cyclohexane	C12 H14	14.902	158.10962	159.1169	-

**Table 4:** Disintegration study on three selected turmeric dietary supplements (DS-3, 8, and 9) performed according to USP 39 general chapters 2040 and 701 for immediate-release DS forms.

Sample	Form	Test Solution Used for Agitation	Temp °C Start	Temp °C Stop	Length of Agitation Time (Minutes)	Num of Pills Disintegrated	Test Result
DS-3	hard-shell capsule	0.05 M Acetate Buffer	35.6	35.9	<30	6	Pass
DS-8	tablet	Water	36.5	36.3	<30	6	Pass
DS-9	hard-shell capsule	0.05 M Acetate Buffer	36.6	36.6	<30	6	Pass

## Data Availability

The original contributions presented in the study are included in the article/Supplementary Material, further inquiries can be directed to the corresponding author.

## Supplementary Materials

The following supporting information can be downloaded at https://www.mdpi.com/article/10.3390/foods14010007/s1, Figure S1: The proportion of the three individual curcuminoids (bisdesmethoxycurcumin (BMC), desmethoxycurcumin (DMC), and curcumin (CUR)) in dietary supplements, powdered turmeric, and ground turmeric rhizomes. Commercial turmeric DS samples included eight capsules (DS-1-DS-7 and DS-9), one tablet (DS-8), three ground turmeric samples (DS-10-DS-12), and three ground turmeric rhizomes (TR-1, TR-2, and TR-3). Figure S2. GCMS analysis of hexane extract of a typical turmeric dietary supplement.
