# Supplementary material for: Comparative Investigation of Untargeted and Targeted Metabolomics in Turmeric Dietary Supplements and Rhizomes
Source: Foods. 2024 Dec 24;14(1):7. doi: 10.3390/foods14010007 (PMC11720146; doi:10.3390/foods14010007)
Supplement: Supplementary file 1 [file foods-14-00007-s001.zip › foods-3344232-supplementary.pdf]

Comparative Investigation of Untargeted and Targeted Metabolomics in Turmeric Dietary Supplements and Rhizomes

Jashbir Singh [1,2], Fakir Shahidullah Tareq [1,3] and Devanand L. Luthria [1,*]

1 Methods and Application of Food Composition Laboratory, Beltsville Human Nutrition Research Center, Ag-ricultural Research Service, U.S. Department of Agriculture, Beltsville, MD 20705, USA

2 Oak Ridge Institute for Science and Education (ORISE) Fellow, Oak Ridge, TN 37831, USA

3 Department of Nutrition and Food Science, College of Agriculture & Natural Resources, University of Maryland, College Park, MD 20742, USA

* Correspondence: dave.luthrial@usda.gov

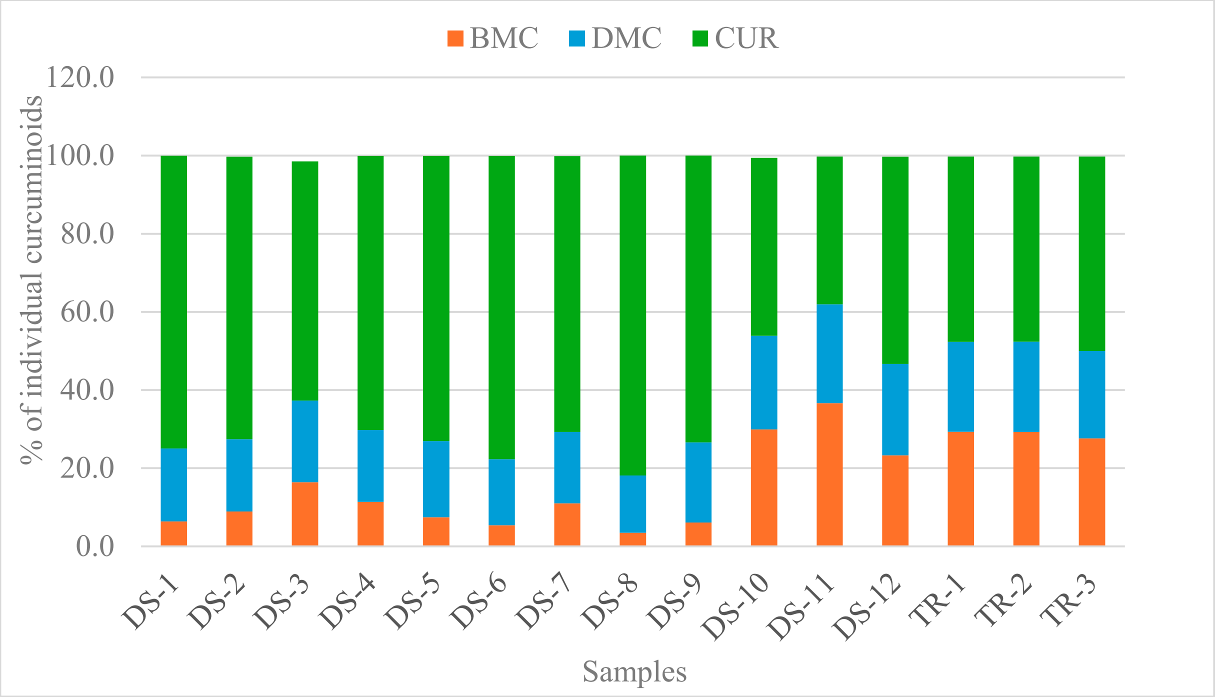

Figure S1. The proportion of the three individual curcuminoids (bisdesmethoxycurcumin (BMC), desmethoxycurcumin (DMC), and curcumin (CUR)) in dietary supplements, powdered turmeric, and ground turmeric rhizome. Commercial turmeric DS; eight capsules (DS-1-DS-7 and DS-9), one tablet (DS-8), three ground turmeric samples (DS-10-DS-12), and three ground turmeric rhizomes (TR-1, TR-2, and TR-3).

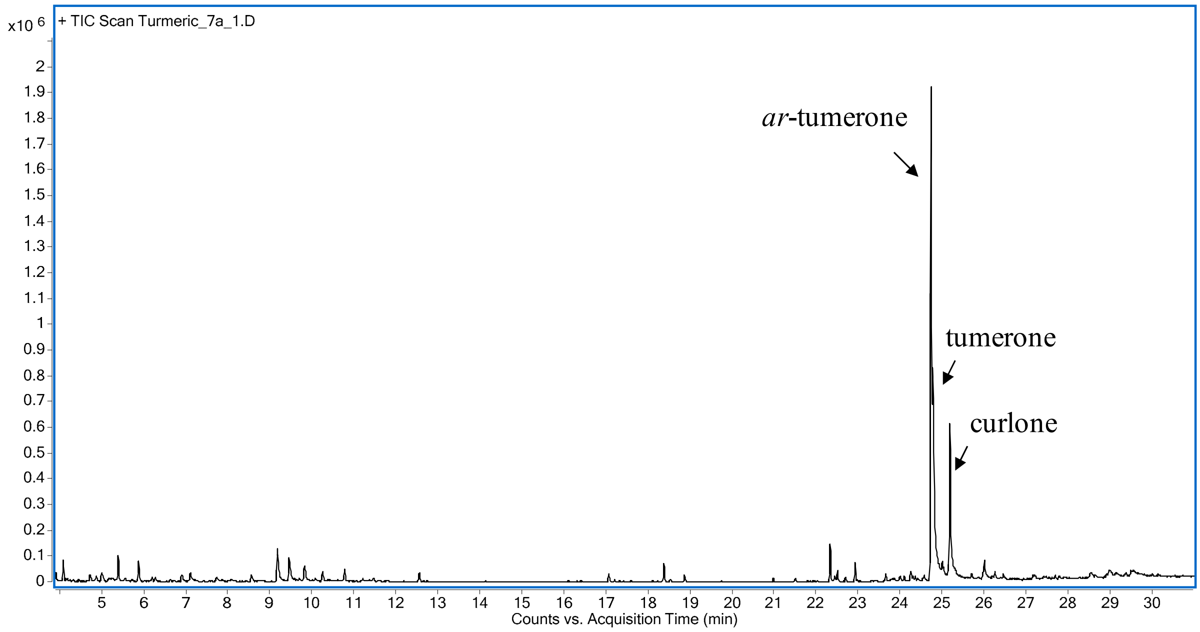

Figure S2. GCMS analysis of hexane extract of a typical turmeric dietary supplement.
